# Precision medication based on the evaluation of drug metabolizing enzyme and transporter functions

**DOI:** 10.1093/pcmedi/pbaf004

**Published:** 2025-02-22

**Authors:** Yanrong Ma, Jing Mu, Xueyan Gou, Xinan Wu

**Affiliations:** The First Clinical Medical College, Lanzhou University, Lanzhou 730000, China; Department of Pharmacy, The First Hospital of Lanzhou University, Lanzhou 730000, China; The First Clinical Medical College, Lanzhou University, Lanzhou 730000, China; The First Clinical Medical College, Lanzhou University, Lanzhou 730000, China; The First Clinical Medical College, Lanzhou University, Lanzhou 730000, China; Department of Pharmacy, The First Hospital of Lanzhou University, Lanzhou 730000, China

**Keywords:** precision medicine, drug metabolizing enzyme, drug transporter, probe, biomarker

## Abstract

Pharmacogenomics, therapeutic drug monitoring, and the assessments of hepatic and renal function have made significant contributions to the advancement of individualized medicine. However, their lack of direct correlation with protein abundance/non-genetic factors, target drug concentration, and drug metabolism/excretion significantly limits their application in precision drug therapy. The primary task of precision medicine is to accurately determine drug dosage, which depends on a precise assessment of the ability to handle drugs *in vivo*, and drug metabolizing enzymes and transporters are critical determinants of drug disposition in the body. Therefore, accurately evaluating the functions of these enzymes and transporters is key to assessing the capacity to handle drugs and predicting drug concentrations in target organs. Recent advancements in the evaluation of enzyme and transporter functions using exogenous probes and endogenous biomarkers show promise in advancing personalized medicine. This article aims to provide a comprehensive overview of the latest research on markers used for the functional evaluation of drug-metabolizing enzymes and transporters. It also explores the application of marker omics in systematically assessing their functions, thereby laying a foundation for advancing precision pharmacotherapy.

## Graphical Abstract

**Graphical Abstract:**
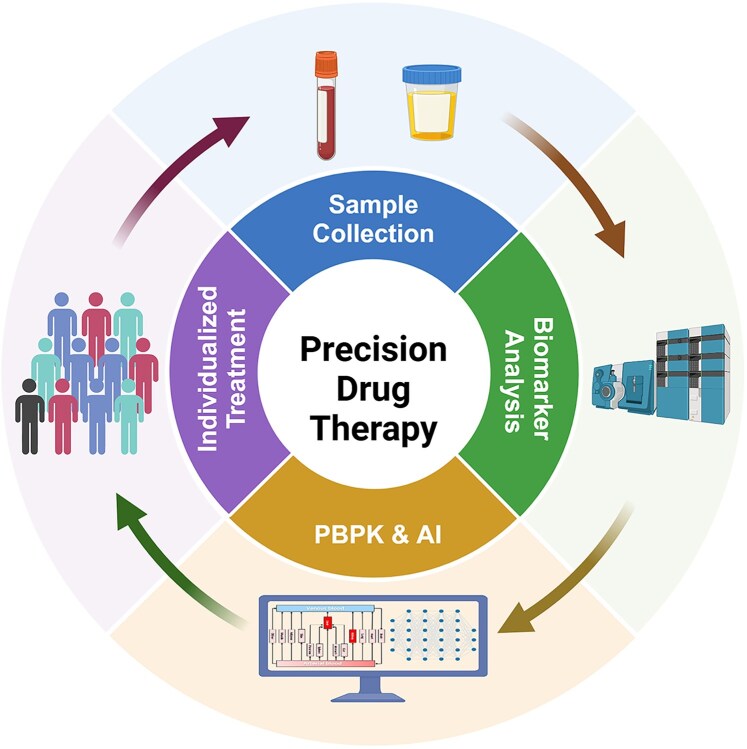
The workflow for precision drug therapy based on the evaluation of drug metabolizing enzyme and transporter functions.

## Introduction

Drugs are the primary tools for the prevention, treatment, and diagnosis of diseases, serving as the foundation for safeguarding public health. While drugs play a therapeutic role, they may also trigger adverse reactions or lead to drug-induced diseases. With the rapid increase in the number of new drugs and improper or excessive use of medications, tens of thousands of patients suffer disabilities or die each year. This has become a serious public health issue, posing a significant challenge to medical safety [1]. According to World Health Organization (WHO) data, ∼50% of patients worldwide are subjected to improper medication use [2]. In the European Union, 5% of all hospitalized patients are admitted due to adverse drug reactions, and ∼197 000 people die each year as a result [3]. In the United States, the incidence of serious adverse drug reactions among hospitalized patients is 6.7% [4]. In 2023, the number of adverse drug reaction reports in China reached 2.023 million, marking 23 consecutive years of growth [5]. The liver and kidneys are key organs responsible for drug metabolism and excretion and are highly susceptible to drug-induced damage. The incidence of drug-induced acute kidney injury ranges from 14% to 26%, while the incidence of kidney injury caused by antibiotics can be as high as 36% [6, 7]. The incidence of drug-induced liver injury ranges from 0.0139% to 0.0191%, with the incidence in China being 0.0238% [8–10]. With the aging population, elderly individuals have become an increasingly large group. Elderly patients often suffer from multiple diseases, making polypharmacy inevitable, and they are also more likely to be on long-term medication. This significantly increases the proportion of improper medication use and the occurrence of adverse drug reactions.

**Figure 1.**
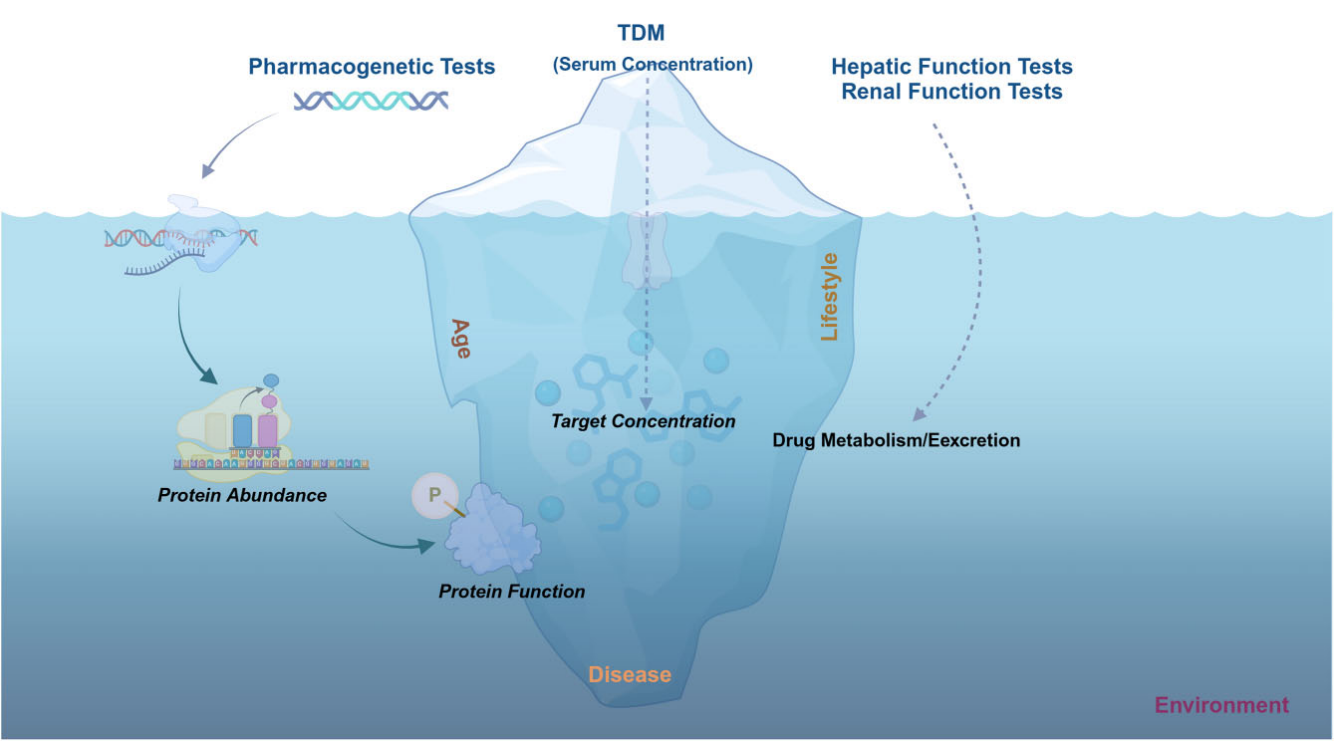
The current status of precision drug therapy. Pharmacogenetic testing, therapeutic drug monitoring (TDM), and hepatic and renal function evaluation systems represent only the tip of the iceberg in precision medicine: pharmacogenetic testing does not reflect the abundance or functionality of drug-related targets, TDM does not indicate the target level of drug, and hepatic and renal function evaluations do not capture the metabolic and excretory capacity of these organs for drugs.

Many factors contribute to the irrational use of medications, but most of these issues can be effectively mitigated through improving patient education, optimizing healthcare service processes, and strengthening drug supply and quality regulation. However, challenges persist due to inaccurate dosing resulting from individual differences, disease states, and drug interactions. It is well-known that most current drug instructions follow a ‘one-size-fits-all’ approach, applying the same prescription to thousands of patients, which lacks precision in dosing. In January 2015, the Obama administration announced the “Precision Medicine Initiative”, aimed at transforming medical research and clinical practice. Precision drug therapy is a crucial component of precision medicine, with its core philosophy being to tailor and implement individualized drug treatment plans based on individual differences of patients (such as genetic background, lifestyle, and environmental factors). Precise dosing not only achieves satisfactory clinical efficacy but also significantly reduces the occurrence of adverse drug reactions and drug-induced diseases. At present, dose adjustments based on pharmacogenetic testing and therapeutic drug monitoring have greatly promoted precision drug therapy. However, pharmacogenetic testing faces numerous issues and challenges. The relationship between genes and drugs is complex, and non-genetic factors (such as disease, age, environment, lifestyle, and protein abundance) also influence drug disposition (Fig. 1). Additionally, for certain genotypes, intra-genotype and inter-genotype variations are comparable [11]. The abundance of various drug metabolizing enzymes can vary up to 20-fold between individuals, with CYP3A4 showing an even greater variation of up to 129-fold among the Chinese population [12, 13]. Although blood drug concentration reflects the extent of an individual's handling of a drug, there are significant limitations to therapeutic drug monitoring, including delays in detection that lead to poor timeliness in adjusting drug dosages, fluctuations in drug concentration due to differences in sampling times, and the fact that blood drug concentration indirectly reflects the concentration at the target site.

Accurate determination of drug dosage relies on a precise understanding of the body's drug disposition capability. Currently, there are no direct indicators for evaluating the body's ability to process drugs in a clinical setting. Instead, indirect organ function indicators are commonly used to reflect the body's drug disposition capacity, such as liver and kidney function indicators. Hepatic function tests (such as alanine aminotransferase, aspartate aminotransferase, alkaline phosphatase, and total bilirubin) can provide some information about liver health, but these biomarkers cannot directly reflect the liver's ability to metabolize drugs (Fig. 1). It is evident in everyday life that even people with normal liver function may have significant differences in alcohol metabolism rates. Additionally, the expression or function of CYP2E1 is significantly increased in patients with non-alcoholic or alcoholic fatty liver disease [14–16]. Similarly, kidney function indicators (such as cystatin C, creatinine, and blood urea nitrogen) primarily reflect glomerular filtration function, while most drugs undergo tubular secretion or reabsorption [17]. For example, creatinine clearance remained within the normal range, but renal excretion of fluconazole (which is primarily excreted unchanged through the kidneys) was significantly reduced in HIV patients [18]; vancomycin is commonly dosed or adjusted in clinical practice based on creatinine clearance, yet only 20% of patients maintain drug concentrations within the therapeutic window [19].

Drug metabolizing enzymes and transporters play a critical role in the absorption, distribution, metabolism, excretion, and toxicity (ADMETox) of drugs. Drugs are taken up into cells by uptake transporters (Phase 0 metabolism), metabolized by enzymes (Phase I and II metabolism), and ultimately expelled from the cells via efflux transporters (Phase III metabolism). The synergistic effect and interplay of drug metabolizing enzymes and transporters determine the pharmacokinetic characteristics and the ultimate therapeutic effects of a drug [20, 21]. The functions of drug metabolizing enzymes and transporters are intricately linked to genetic factors, protein abundance, and other influencing variables. Functional evaluation can integrate all these aspects, enabling predictions prior to drug therapy and effectively overcoming the limitations of genetic testing, therapeutic drug monitoring, and organ function assessments.

**Figure 2.**
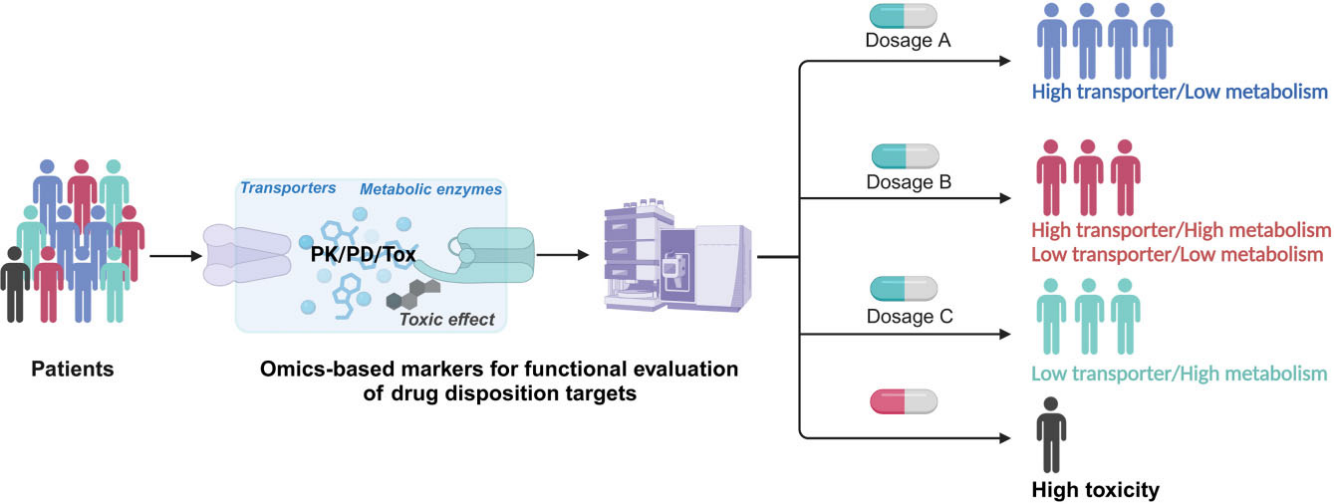
Precision drug therapy based on functional evaluation omics of drug-related targets. By assessing the levels of exogenous probes or endogenous biomarkers for drug transporters, metabolic enzymes, and toxicity targets, this approach enables accurate classification of drug disposition capacity *in vivo*, allowing precise adjustments in drug dosing. In this illustration, the drug target in the liver is subject to metabolic inactivation.

Omics-based markers for functional evaluation of drug disposition targets represents a multi-level systems biology approach that focuses on assessing the functional roles of drug metabolism, transport, and toxicity-related targets. Integrating this framework with systems pharmacology methods aims to provide a precise evaluation of the *in vivo* drug disposition process and the functional status of associated targets (Fig. 2). Although research in this area is still in its exploratory phase, it faces three major challenges: first, how to evaluate the function of drug disposition targets; second, how to apply systems biology methods to reveal drug–target interactions; and third, how to use functional evaluation omics data to simulate and predict the dynamic process of drugs in the body. Currently, some progress has been made in the evaluation of drug-metabolizing enzyme and transporter functions based on exogenous probes and endogenous biomarkers. This is of great significance for understanding the *in vivo* disposition of drugs and optimizing therapeutic strategies [22–28].

## Drug metabolizing enzymes and functional evaluation markers

### Drug metabolizing enzymes

Phase I and Phase II metabolic enzymes play essential roles in drug metabolism. Phase I enzymes are responsible for the preliminary metabolism of drugs, primarily through oxidation, reduction, and hydrolysis reactions, which convert drugs into metabolites with stronger polarity. Among Phase I enzymes, cytochrome P450 (CYP) is a key enzyme system in humans, governing the metabolism of ∼80% of clinically used drugs. CYPs consist of 57 different enzymes in humans, categorized into 18 families and 43 subfamilies based on the similarity of their amino acid sequences. In the CYPs, the sulfur atom of the cysteine (Cys) residue in the L-helix preceding the loop region binds to the heme iron, forming a Cys–Fe bond. The catalytic core consists of the heme group, which is surrounded by the I and L helices. The catalytic domain is connected to the NH_2_-terminal transmembrane helix of CYPs via a proline-rich region, while the substrate-binding site is located at the distal end of the heme. The secondary structure of mammalian CYPs is relatively conserved, consisting of 12 α-helices (A–L) and 4 β-sheets (1–4). Notably, the B′ helix, along with the F and G helices, exhibits significant sequence and structural variability among different CYPs, which is more conducive to catalyzing drugs with different structures. CYPs contain additional helices, such as the F′ and G′ helices, the F and G helices, and the F-G loop. These structures are crucial for facilitating substrate entry into the active site [29].

In CYPs, CYP1A2, CYP2D6, CYP2C9, CYP2C19, and CYP3A4 are essential in clinical drug metabolism, metabolizing ∼8.9%, 20%, 12.8%, 6.8%, and 30.2% of drugs, respectively [30]. In Japanese liver microsomes, the protein content ranking is CYP2C9 > CYP3A4 > CYP2E1 > CYP2A6 > CYP2C8 [12], whereas in Chinese individuals, the order is CYP2E1 > CYP2C9 > CYP3A4 > CYP1A2 > CYP3A [13]. CYPs play an essential role in the metabolism of endogenous substances, such as hormones, lipids, and vitamins. In addition to classical CYP enzymes, non-classical Phase I metabolic enzymes also contribute significantly to drug metabolism. These enzymes mainly include flavin-containing monooxygenases, aldehyde oxidases, xanthine oxidases, carbonic anhydrases, monoamine oxidases, and peptidases.

Phase II metabolic enzymes are responsible for the second phase of drug metabolism. These enzymes facilitate conjugation reactions, further transforming polar metabolites generated by Phase I metabolism into more water-soluble compounds that are easier to excrete. Phase II enzymes play a critical role in detoxification and elimination of drugs. The primary Phase II metabolic enzymes include uridine 5'-diphospho-glucuronosyltransferase (UGT), sulfotransferases, glutathione *S*-transferases, *N*-acetyltransferases, methyltransferases, and amino acid conjugation enzymes [31].

### Exogenous probes and endogenous biomarkers for drug metabolizing enzymes

Exogenous probes are typically selected because they produce specific metabolites under the action of a particular drug-metabolizing enzyme without being affected by other enzymes. By measuring the metabolites of these probe drugs or the ratio of the parent drug to its metabolites, the function of a specific enzyme can be accurately reflected. Exogenous probes are highly responsive to small metabolic changes, and by quantifying changes in the parent drug and its metabolites, they can sensitively reflect subtle variations in enzyme activity. Many exogenous probes have undergone extensive safety evaluations and have well-established pharmacokinetics and pharmacodynamics. Probe drugs are usually administered at low doses, minimizing potential risks for the participants while still providing valuable metabolic information. Currently, the activity of a single enzyme or multiple enzymes can be assessed by administering a single exogenous probe or a “cocktail” of probes. The cocktail approach enables the simultaneous evaluation of multiple enzymes without significant interactions between probes, providing a comprehensive assessment of enzyme activity. In contrast, endogenous biomarkers offer a non-invasive alternative, effectively avoiding potential side effects, immune reactions, and ethical or regulatory concerns. As a result, endogenous biomarkers are widely accepted in clinical practice. However, endogenous biomarkers may have limitations, including complex metabolic pathways, lack of specificity, high background noise, large variability, and challenges in determining their dynamic range. Nevertheless, endogenous biomarkers remain valuable for evaluating enzyme function, particularly in scenarios where invasive methods are impractical or real-time physiological enzyme activity needs to be assessed.

#### CYP1A2

CYP1A2 is primarily expressed in the liver, and its metabolism mainly occurs through oxidation reactions, including hydroxylation, demethylation, and *N*-oxidation. It mediates the metabolism of drugs such as phenacetin, caffeine, theophylline, clozapine, olanzapine, imipramine, mirtazapine, propranolol, and duloxetine. Smoking, certain foods (such as cruciferous vegetables), and drugs (such as omeprazole) can induce the expression of CYP1A2, while its activity is significantly reduced in patients with inflammation or cholestasis [32]. Fluvoxamine, ciprofloxacin, and cimetidine are strong inhibitors of CYP1A2. Genetic polymorphisms can significantly influence the function of CYP1A2, with the CYP1A21F allele associated with higher activity and the CYP1A21C allele linked to lower activity [33]. The exogenous probe recommended by the U.S. Food and Drug Administration (FDA) is phenacetin, which undergoes the *O*-deethylation mediated by CYP1A2 to produce acetaminophen (Table 1), with a *K*_m_ ranging from 1.7 to 152 μM [34]; 7-ethoxyresorufin is also recommended for evaluating CYP1A2 function *in vitro*. In addition, caffeine, theophylline, and tacrine are also accepted for assessing CYP1A2 function (Table 1) [34–36], but *in vivo* studies have shown that tacrine is not an ideal probe for this purpose [37].

**Table 1.** Exogenous and endogenous probes for CYPs.

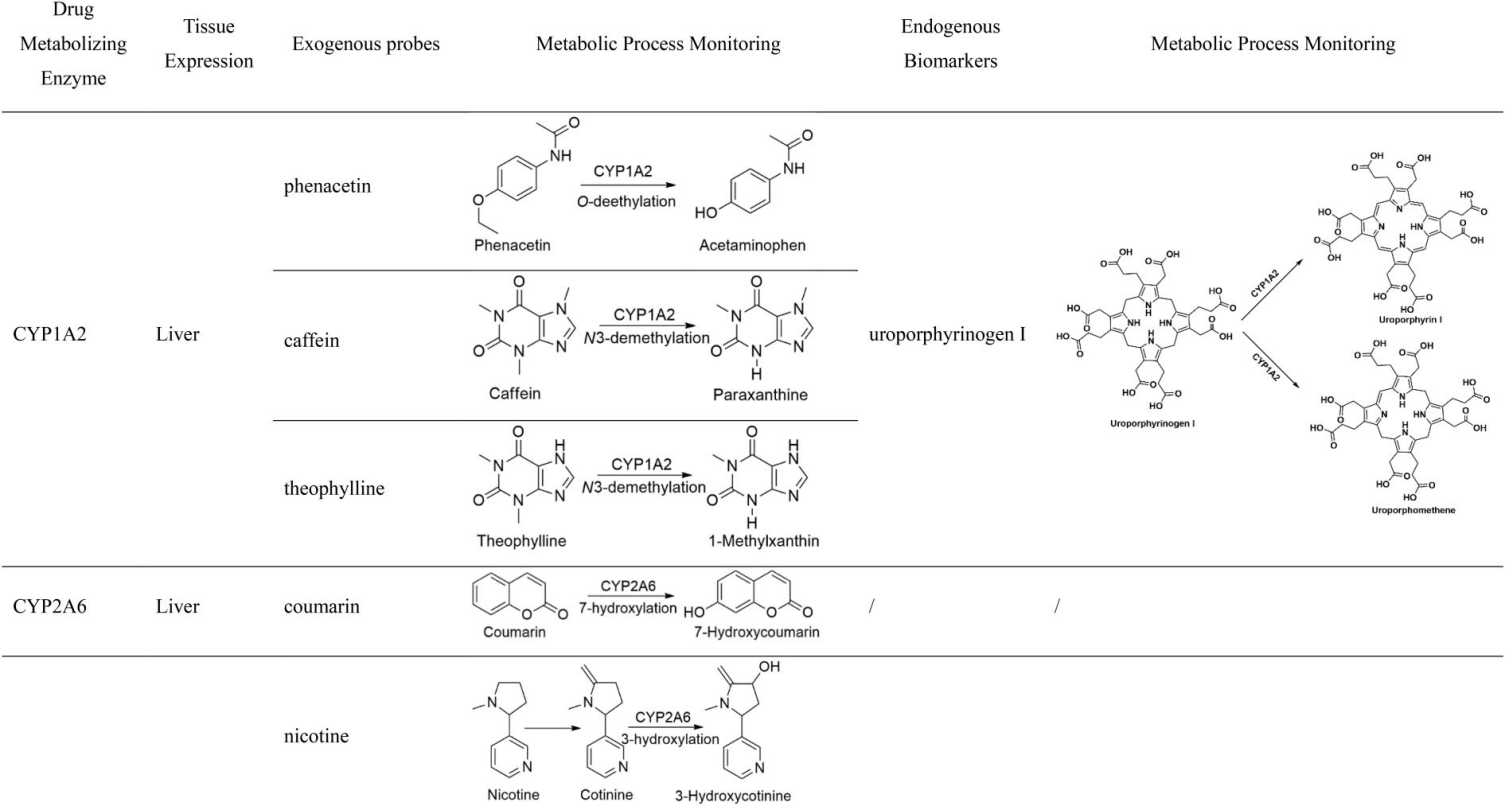
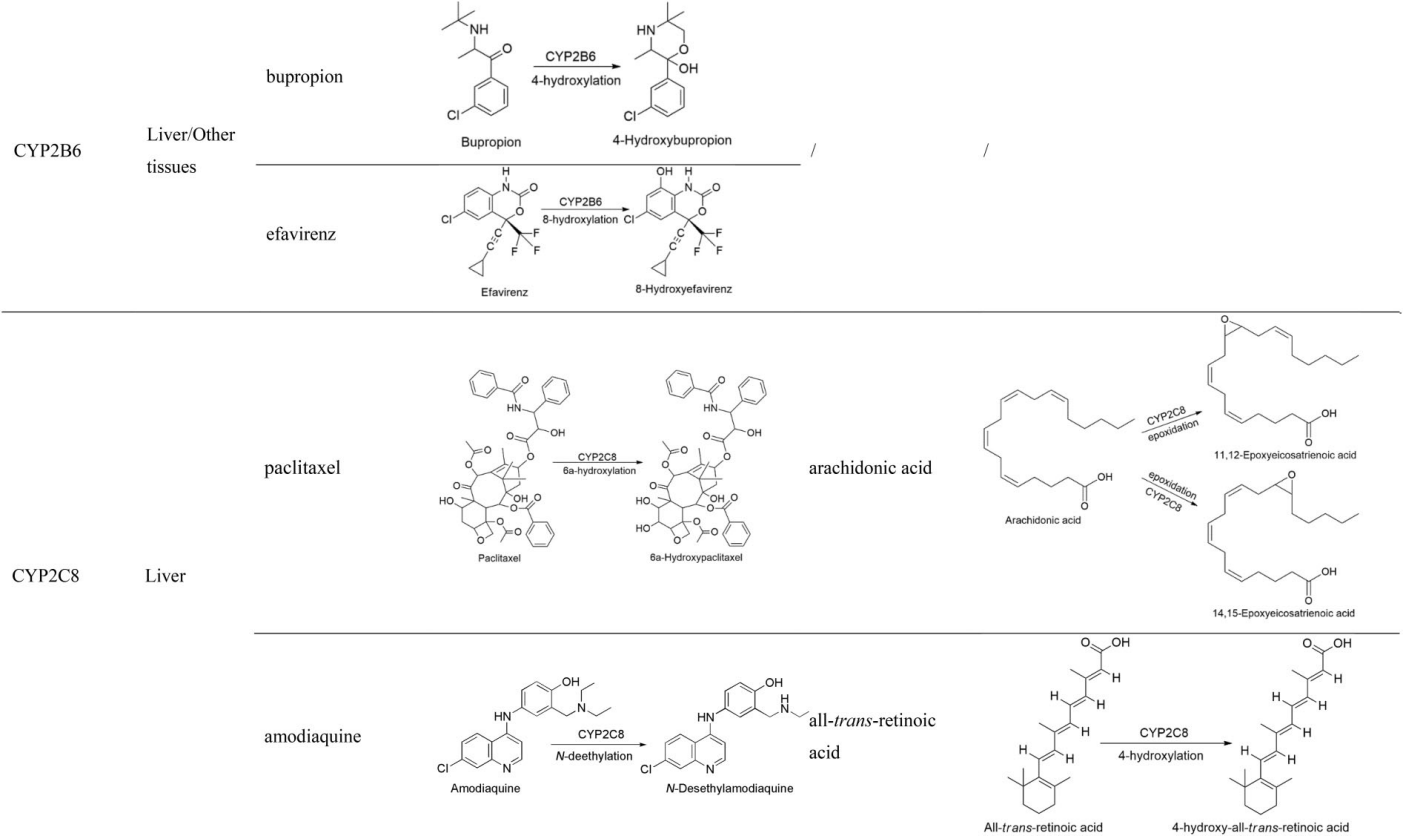
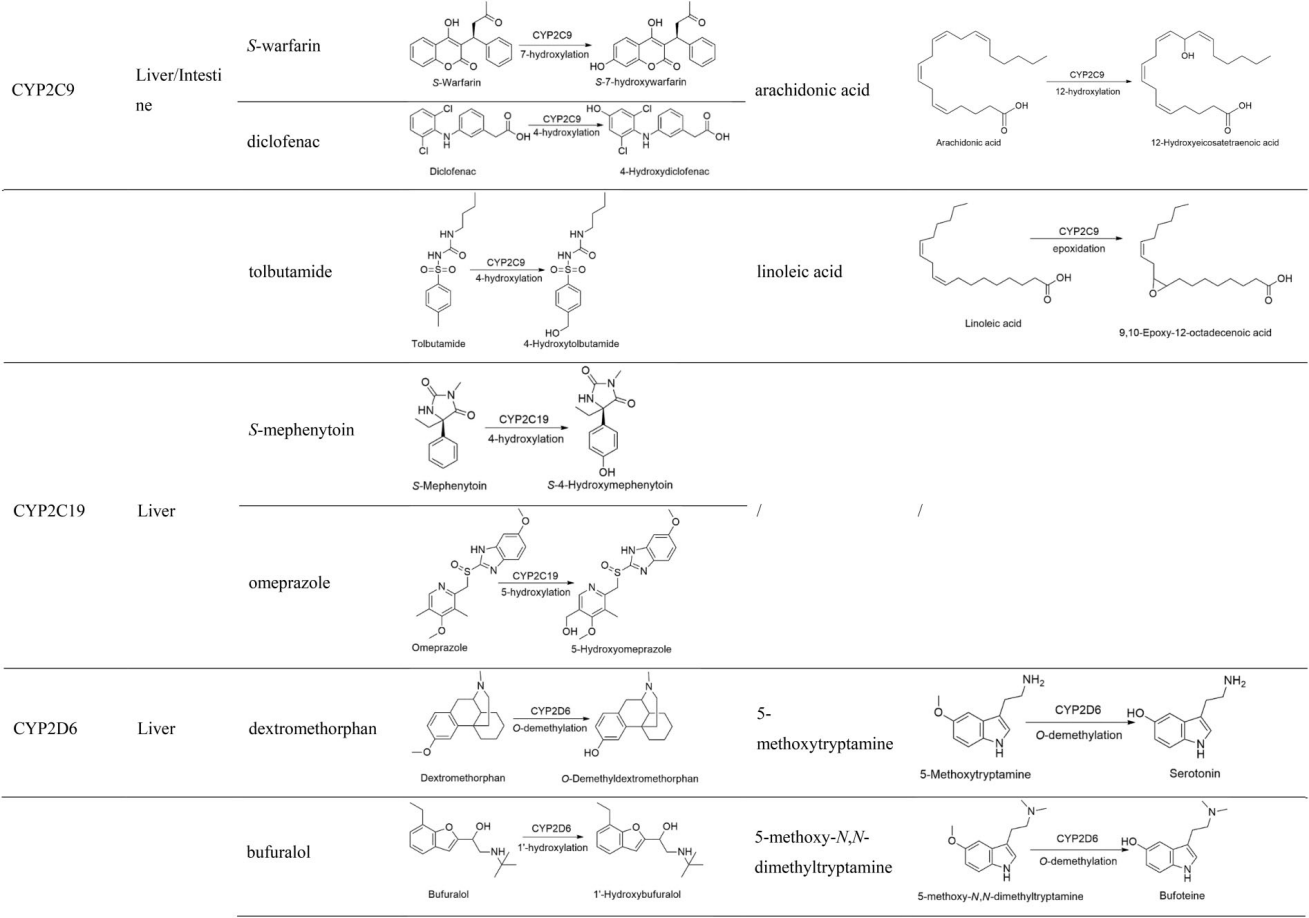
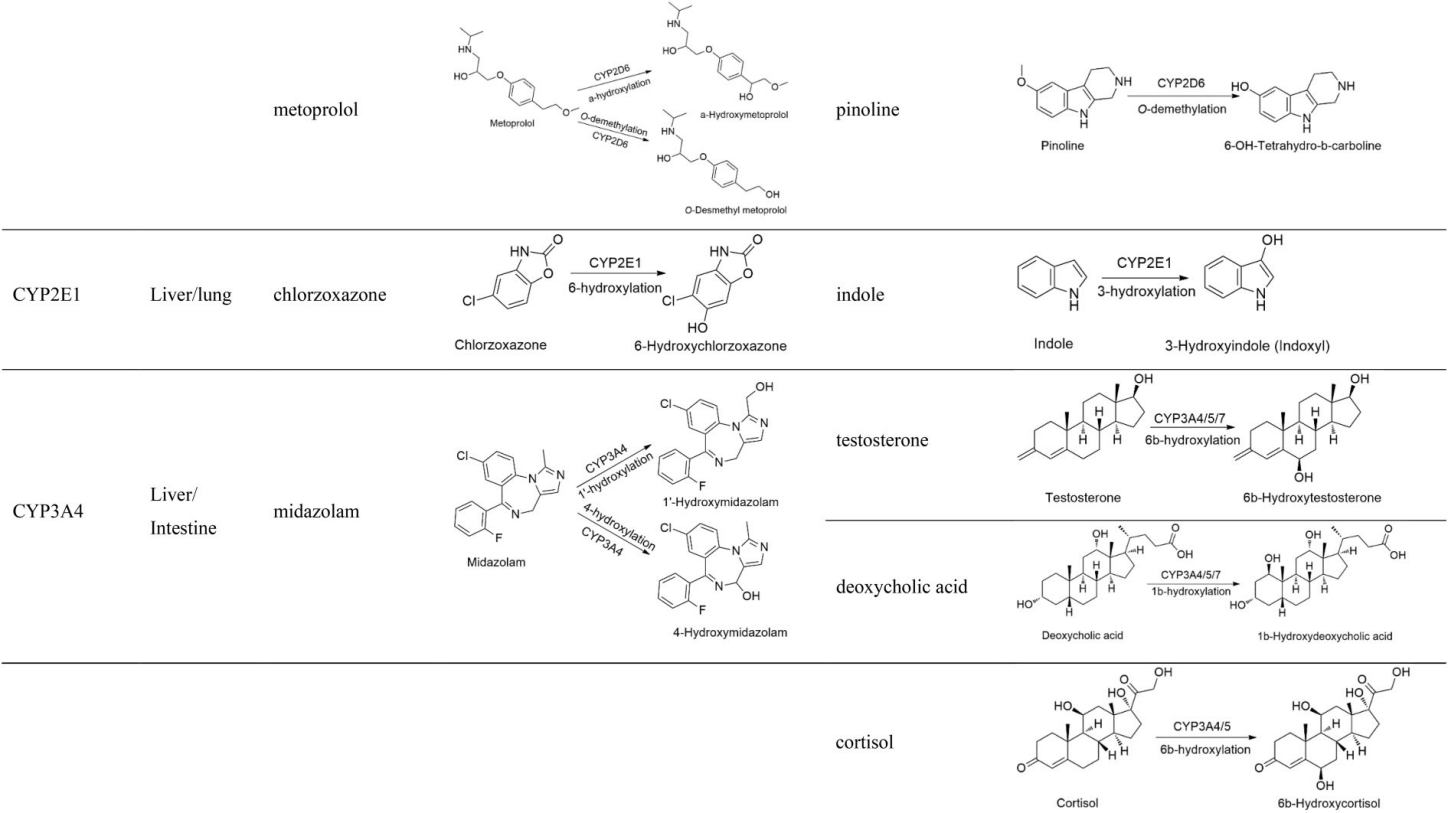

CYP1A2 is involved in the metabolism of several endogenous substances, including progesterone, estrogen, estradiol-3-methyl ether, melatonin, all-*trans*-retinol, all-*trans*-retinal, linoleic acid, uroporphyrinogen, and phosphatidylcholine [38]. CYP1A1 mediates the conversion of progesterone to 16α-/6β-hydroxyprogesterone, while CYP1A2 is also involved in the formation of 6β-hydroxyprogesterone [39]. Estrone is hydroxylated at the 2/4/16α site by CYP1A2, while CYP1A1 catalyzes the oxidation of estrone at the C10 site to form a quinone [40]. The hydroxylation sites for estradiol are at C2 and C4, with CYP1A1 primarily mediating the formation of 2-hydroxyestradiol, as well as facilitating 6α-/15α-hydroxylation and quinone formation [40]; CYP1A2 catalyzes hydroxylation at C2, C4, and C16, with C2-hydroxyestradiol being the primary hydroxylated metabolite produced by CYP1A2 [38, 41]. Progesterone and estrogen are metabolized by multiple enzymes to produce various metabolites, and their potential as biomarkers for evaluating CYP1A function requires further confirmation. Melatonin is converted into 6-hydroxymelatonin by CYP1A1, CYP1A2, and CYP1B1, with CYP1A2 being the primary enzyme involved. CYP1A2 can also catalyze the *O*-demethylation of melatonin to produce *N*-acetylserotonin [42]. Although CYP1A2 is a key enzyme in melatonin metabolism in the liver, the widespread expression of CYP1B1 in extrahepatic tissues may influence the reliability of the ratio of 6-hydroxymelatonin to melatonin as a biomarker for CYP1A2 function [42]. All-*trans*-retinol is converted into all-*trans*-retinoic acid through two-step metabolism by CYP1A1 and CYP1A2. The first metabolic step, a rate-limiting process, converts all-*trans*-retinol to all-*trans*-retinal, with both CYP1A1 and CYP1A2 exhibiting comparable metabolic capacities. Linoleic acid, which plays an important role in physiological processes, cannot be synthesized in the body and is primarily obtained through dietary intake. The metabolism of linoleic acid involves various enzymes, with CYP1A2 mediating the formation of 13-hydroxyoctadecdienoic acid and 9,10/12,13-*cis*-epoxyoctadecenoic acid [43]. CYP1A2 specifically catalyzes the conversion of uroporphyrinogen I into uroporphyrin I, but its metabolic activity in humans is relatively low. Additionally, CYP1A2 can catalyze the production of uroporphomethene through iron-dependent oxidation (Table 1) [44–46]. Despite the wide range of endogenous substances metabolized by CYP1A2, it remains uncertain whether they can serve as reliable biomarkers for evaluating its function. However, the metabolism of uroporphyrinogen by CYP1A2 shows potential due to its specificity.

#### CYP2A6

CYP2A6 is primarily expressed in the liver and plays an important role in the metabolism of various drugs and environmental toxins, particularly nicotine, aflatoxin B1, and nitrosamines. Substrate drugs include letrozole, tegafur, and citrate. CYP2A6 exhibits extensive genetic polymorphisms, with significantly reduced activity in many genotypes. The FDA-recommended exogenous probe for CYP2A6 is coumarin, which is metabolized to 7-hydroxycoumarin by CYP2A6 (Table 1) with a *K*_m_ ranging from 0.3 to 2.3 μM, and its effects on other CYPs are negligible [34]. Nicotine is metabolized to cotinine in humans, which is further metabolized by CYP2A6 into 3-hydroxycotinine. The ratio of 3-hydroxycotinine to cotinine can be used to evaluate CYP2A6 function (Table 1) [47].

CYP2A6 is involved in the oxidative metabolism of endogenous steroid hormones, but the metabolic pathways are not yet well defined. Additionally, steroid hormones undergo extensive metabolic processes *in vivo*, and this lack of specificity may limit their use as endogenous biomarkers for evaluating CYP2A6 function [48].

#### CYP2B6

CYP2B6 is primarily expressed in the liver, accounting for only 2%–5% of total P450s, and its expression can vary by as much as 300-fold among individuals [30]. In fetuses, its expression is very low, with ∼36% showing undetectable levels [49]. CYP2B6 exhibits extensive genetic polymorphisms, with increased activity in CYP2B6*4A, and decreased activity in CYP2B6*6A, CYP2B6*16, and CYP2B6*26. Substrate drugs for CYP2B6 include bupropion, efavirenz, methadone, lidocaine, propofol, mephenytoin, artemether, artemisinin, nevirapine, and pethidine [50]. The FDA-recommended exogenous probes are bupropion and efavirenz, which are hydroxylated by CYP2B6 to form 4-hydroxybupropion and 8-hydroxyefavirenz, respectively (Table 1), with *K*_m_ values of 67–168 μM and 12.4 μM [34, 51]. A small amount of bupropion can also be converted into *erythro*- and *threo*-dihydrobupropion via non-P450-dependent enzymes [52]. *S*-Efavirenz is primarily metabolized by CYP2B6 into *S*-8-hydroxyefavirenz, and to a lesser extent by CYP2A6 into *S*-7-hydroxyefavirenz. *S*-8-Hydroxyefavirenz can further be metabolized to *S*-8,14-dihydroxyefavirenz by CYP2B6 [53].

CYP2B6 is also involved in the metabolism of steroid hormones, mediating the 2-hydroxylation of 17β-estradiol, although its metabolic activity in this process is relatively weak [54]. CYP2B6 also catalyzes the 16β-hydroxylation of testosterone, but CYP2C8, CYP2C19, CYP3A4, and CYP3A17 also contribute to this process [55]. Therefore, endogenous biomarkers have not yet been identified to specifically evaluate CYP2B6 function.

#### CYP2C8

CYP2C8 is highly expressed in the liver and shares 74% sequence homology with CYP2C9. It mediates the metabolism of various drugs, including paclitaxel, amodiaquine, cerivastatin, daprodustat, dasabuvir, enzalutamide, montelukast, pioglitazone, and repaglinide. In addition, CYP2C8 is also involved in lipid metabolism and steroid metabolism [56]. The FDA-recommended exogenous probes are paclitaxel and amodiaquine, which are metabolized by CYP2C8 into 6α-hydroxypaclitaxel and *N*-deethylamodiaquine, respectively (Table 1), with *K*_m_ values of 5.4–19 μM and 0.9–1.2 μM [34, 57]. The formation of 6α-hydroxypaclitaxel is dependent on CYP2C8, with minimal contributions from CYP2C9, CYP2C19, and CYP3A. Additionally, low concentrations of paclitaxel (10 μM) have little effect on other CYPs [58, 59]. CYP2C8 has a high affinity for amodiaquine. However, CYP2C9, CYP2D6, and CYP3A4 may also significantly contribute to this process, and amodiaquine has a strong inhibitory effect on the activity of CYP2D6 and CYP2C9 [60, 61].

CYP2C8 is involved in the metabolism of endogenous substances such as steroid hormones, retinoic acid, and arachidonic acid. *In vitro*, 17β-estradiol and estrogens are substrates of CYP2C8, which mediates the hydroxylation at the 2- and 4-sites of 17β-estradiol, but their contributions are limited [62]. Arachidonic acid, as an endogenous substrate of CYP2C8, is primarily epoxidized into epoxyeicosatrienoic acids (EETs), including 11,12- and 14,15-EETs (Table 1). However, these epoxide metabolites are further converted into dihydroxyeicosatrienoic acids by soluble epoxide hydrolase [63]. CYP2C8 is also a key enzyme for the 4-hydroxylation of all-*trans*-retinoic acid (Table 1). While CYP3A4 is involved in this metabolic process, its contribution is relatively minor [64]. Therefore, arachidonic acid and all-*trans*-retinoic acid may serve as endogenous biomarkers for evaluating CYP2C8 function.

#### CYP2C9

CYP2C9 constitutes ∼20% of hepatic P450 proteins and is also expressed in the intestine. It is involved in the oxidation of various drugs, including *S*-warfarin, irbesartan, losartan, celecoxib, diclofenac, ibuprofen, indomethacin, piroxicam, glibenclamide, gliclazide, glimepiride, ethinylestradiol, and phenytoin. Several mutations exist in CYP2C9, of which CYP2C9*2 and CYP2C9*3 significantly reduce its activity [65, 66]. The FDA-recommended exogenous probes are *S*-warfarin and diclofenac, which are metabolized by CYP2C9 into *S*-7-hydroxywarfarin and 4-hydroxydiclofenac, respectively (Table 1), with *K*_m_ values of 4.0 μM and 3.4–52 μM [34, 67]. Warfarin can be hydroxylated at different positions by various CYPs, but *S*-warfarin is primarily metabolized (>80%) by CYP2C9 into *S*-7-hydroxywarfarin and *S*-6-hydroxywarfarin, with *S*-7-hydroxywarfarin being the predominant metabolite in human plasma [67, 68]. A small amount of *S*-warfarin can also be metabolized by CYP2C8, CYP2C18, CYP2C19, and CYP3A4 into *S*-4-, *S*-8-, and *S*-10 (*R, S*)-hydroxywarfarin [69]. Diclofenac is used solely for activity measurement of CYP2C9 *in vitro*, with its primary metabolite being 4-hydroxydiclofenac, along with small amounts of 3-hydroxydiclofenac and 5-hydroxydiclofenac [70]. *In vitro* studies have shown that the 4-hydroxylation and 3-hydroxylation of diclofenac are primarily mediated by CYP2C9, while the 5-hydroxylation is mediated by CYP3A4 and other CYP2Cs (CYP2C8, CYP2C18, and CYP2C19) [71, 72]. Some studies demonstrated that CYP2C8 and CYP2C19 could participate in the formation of 4-hydroxydiclofenac. Additionally, tolbutamide is metabolized by CYP2C9 into 4-hydroxytolbutamide and can also be used as a probe for measuring CYP2C9 activity *in vitro* and *in vivo* [73, 74].

CYP2C9 is involved in the metabolism of endogenous substances such as arachidonic acid and linoleic acid. CYP2C9 mediates the epoxidation of arachidonic acid and the formation of 12-hydroxyeicosatetraenoic acid, and its contribution to the epoxidation process is smaller compared to CYP2C8, while its contribution to the hydroxylation process is greater (Table 1) [75]. Additionally, the epoxidation of linoleic acid to produce 9,10-epoxy-12-octadecanoate is primarily mediated by CYP2C9 [76]. Therefore, the 12-hydroxylation of arachidonic acid and the epoxidation of linoleic acid may serve as potential biomarkers for evaluating CYP2C9 function.

#### CYP2C19

CYP2C19 is primarily expressed in the liver and mediates the metabolism of various drugs, including proton pump inhibitors, antidepressants, phenytoin, clopidogrel, diazepam, and voriconazole [77]. The CYP2C19*2 and *3 alleles are associated with reduced enzyme activity, whereas the CYP2C19*17 allele leads to increased enzyme activity [77, 78]. The FDA-recommended exogenous probe is *S*-mephenytoin, which is metabolized by CYP2C19 into *S*-4-hydroxymephenytoin (Table 1) with a *K*_m_ of 13–25 μM [34]. *S*-Mephenytoin, at concentrations <50 μM, does not influence the activity of other CYP enzymes. [79]. Omeprazole is recognized as an alternative probe for evaluating CYP2C19 activity. It is metabolized into sulfoxide and hydroxylated products by CYP3A4 and CYP2C19, respectively. The *S*-enantiomer is primarily catalyzed by CYP2C19 to form 5-*O*-desmethyl omeprazole, while the *R*-enantiomer is mainly metabolized by CYP2C19 to produce 5-hydroxy omeprazole and small amounts of 5-*O*-desmethyl omeprazole. Among these metabolic pathways, CYP2C19-mediated hydroxylation is the major metabolic route for omeprazole (Table 1) [80].

CYP2C19 is involved in the biotransformation of steroid hormones, fatty acids, neurotransmitters, and melatonin, but its specificity and contribution to the metabolism of these substances have not yet been fully elucidated [42].

#### CYP2D6

CYP2D6 is predominantly expressed in the liver and mediates the metabolism of drugs such as fluoxetine, paroxetine, amitriptyline, propafenone, metoprolol, dextromethorphan, and codeine. CYP2D6 activity varies greatly among individuals, partly due to its genetic polymorphism. Therefore, accurately assessing CYP2D6 function is crucial for the safe use of its substrate drugs. The FDA-recommended exogenous probes are dextromethorphan and bufuralol, which are metabolized by CYP2D6 into *O*-demethyldextromethorphan and 1-hydroxy bufuralol, respectively (Table 1), with *K*_m_ values of 0.44–8.5 μM and 9–15 μM [34]. The formation of *O*-demethyldextromethorphan shows biphasic enzyme kinetics, with CYP2D6 primarily mediating at low dextromethorphan concentrations, and both CYP2D6 and CYP2C9 contributing at higher concentrations [81]. Dextromethorphan has a negligible effect on other CYPs at concentrations <25 μM [34]. Similarly, the formation of 1-hydroxy bufuralol also follows biphasic enzyme kinetics, being mediated by CYP2D6 at low concentrations and by CYP1A2, CYP2C8, CYP2C9, and CYP2C19 at higher concentrations [81–83]. The risk of affecting CYPs is significantly reduced when bufuralol is used at concentrations <20 μM [34]. Metoprolol is metabolized by CYP2D6 to form α-hydroxymetoprolol and *O*-demethylmetoprolol, and the latter is further metabolized into metoprolol acid [84]. Metoprolol can serve as a probe *in vivo*, with CYP2D6 preferentially metabolizing *R*-metoprolol into *O*-demethylmetoprolol [85].

CYP2D6 mediates the metabolism of endogenous substances such as neurotransmitters, indoleamines, and steroid hormones [86]. CYP2D6 catalyzes the conversion of 4-methoxyphenethylamine into tyramine, which is further hydroxylated to form dopamine. However, these *O*-demethylation and hydroxylation processes exhibit low affinity (*K*_m_ > 55 mM), indicating the limited role of CYP2D6 in the metabolism of these substances [87]. The research team led by Gonzalez identified 5-methoxytryptamine, 5-methoxy-*N,N*-dimethyltryptamine, and pinoline as high-affinity substrates of CYP2D6. Recombinant enzyme studies have confirmed that CYP2D6 mediates the *O*-demethylation of 5-methoxytryptamine, 5-methoxy-*N,N*-dimethyltryptamine, and pinoline with high catalytic activity, while no metabolic activity was observed for other CYPs (Table 1) [88–90]. Therefore, 5-methoxytryptamine, 5-methoxy-*N,N*-dimethyltryptamine, and pinoline hold potential as endogenous biomarkers for evaluating CYP2D6 function.

#### CYP2E1

CYP2E1 is highly expressed in the liver and is involved in the metabolism of drugs and toxins such as ethanol, *p*-nitrophenol, *N*-nitrosodimethylamine, acetaminophen, and chlorzoxazone. It plays a crucial role in oxidative stress, inflammation, and liver diseases [91, 92]. Due to the potential carcinogenicity of *p*-nitrophenol and *N*-nitrosodimethylamine, they can only be used for *in vitro* metabolic activity measurements. Chlorzoxazone is a probe for CYP2E1 *in vivo* (Table 1), with a *K*_m_ of 39–157 μM [34]. The metabolism of chlorzoxazone to 6-hydroxychlorzoxazone exhibits biphasic enzyme kinetics, and it is mediated by both CYP1A2 (*K*_m_ = 3.8 μM) at low concentrations and CYP2E1 (*K*_m_ = 410 μM) at higher concentrations [93].

CYP2E1 is involved in the metabolism of endogenous substances such as acetone, indole, and fatty acids. The 3-hydroxylation of indole to form 3-hydroxyindole is predominantly mediated by CYP2E1 (Table 1) [94–96].

#### CYP3A4

CYP3A4 is the most important enzyme in the P450 superfamily, is responsible for the metabolism of more than half of all drugs, and is highly expressed in the liver and small intestine. The catalytic reactions of CYP3A4 include oxidation, dealkylation, deamination, and hydroxylation. CYP3A4 activity is influenced by various factors, including genetics, diet, medications, and diseases. The FDA currently recommends midazolam as a specific probe for CYP3A4 *in vivo* and *in vitro*. At low concentrations, midazolam primarily generates 1′-hydroxymidazolam (*K*_m_ = 5.6 μM), while at higher concentrations, the formation of 4-hydroxymidazolam increases to 35% (*K*_m_ = 96.9 μM) (Table 1) [97–99]. Midazolam has minimal effect on other CYPs at concentrations <5 μM, however, at higher concentrations, it can significantly inhibit the activity of CYP2B6 and CYP2C8 [34].

CYP3A4 is involved in the metabolism of numerous endogenous substances, including bile acids, cholesterol, steroid hormones, fatty acids, cortisol, melatonin, and vitamin D. Therefore, potential endogenous biomarkers for assessing CYP3A4 function could be derived from these substances. Currently, the FDA recommends testosterone as an endogenous biomarker for CYP3A4, which is metabolized via 6β-hydroxylation to form 6β-hydroxytestosterone (Table 1). Three hydroxylated metabolites of testosterone have been identified in the recombinant CYP3A enzymes: 6β-hydroxytestosterone, 2β-hydroxytestosterone, and 2α-hydroxytestosterone. The contribution of CYP3A5 to the formation of 6β-hydroxytestosterone is comparable to or lower than that of CYP3A4, while the contribution of CYP3A7 is significantly lower than that of CYP3A4. CYP3A5 expression is much lower than that of CYP3A4 in human liver, making its contribution to 6β-hydroxytestosterone formation limited [100]. The formation of 2α-hydroxytestosterone is primarily mediated by CYP3A7. Thus, both 2α-hydroxytestosterone and 6β-hydroxytestosterone can be used as endogenous biomarkers for CYP3A7 [101, 102]. Cholesterol, a key substrate for bile acid and steroid hormone biosynthesis, is converted into 4β-hydroxycholesterol (but not 4α-hydroxycholesterol) by CYP3A4/5. It remains uncertain whether its metabolic rate can serve as a reliable indicator of CYP3A function [103–105]. Deoxycholic acid is metabolized by CYP3A4, CYP3A5, and CYP3A7 to form 1β-hydroxydeoxycholic acid, and the ratio of 1β-hydroxydeoxycholic acid to deoxycholic acid in urine can be used to assess CYP3A activity (Table 1) [106–108]. Cortisol is metabolized to 6β-hydroxycortisol by CYP3A4/5, making the ratio of 6β-hydroxycortisol to cortisol in blood or urine a potential endogenous biomarker for assessing CYP3A activity (Table 1) [109–111].

## Drug transporter and functional evaluation markers

### Drug transporters

Drug transporters are a class of proteins expressed on the cell membrane that can be divided into two major families: the solute carrier (*SLC*) family and the ATP-binding cassette (*ABC*) family. In humans, the *SLC* family consists of ∼350 transporters and the *ABC* family includes 48 transporters. Based on sequence homology, these transporters are further subdivided into various subfamilies. The *SLC* family includes NTCP (*SLC10A1*), PEPT1/2 (*SLC15A1/2*), OCT1/2 (*SLC22A1/2*), OAT1/2/3 (*SLC22A6/7/8*), CNT1/2 (*SLC28A1/2*), ENT1/2 (*SLC29A1/2*), MATE1/2-K (*SLC47A1/2-K*), OATP1B1/1B3 (*SLCO1B1/1B3*), and OATP4C1 (*SLCO4C1*). The *ABC* family includes P-gp (*ABCB1*), BSEP (*ABCB11*), MRP2/3/4 (*ABCC2/3/4*), and BCRP (*ABCG2*). In the intestine, transporters predominantly expressed include OATP1A2/2B1, PEPT1, P-gp, BCRP, and MRP2. In the liver, key transporters are OCT1, OAT2, OATP1B1/1B3, NTCP, P-gp, BCRP, BSEP, and MRP2/3/4. In the kidney, the primary transporters include OCT2, OAT1/2/3, PEPT1/2, OATP4C1, P-gp, BCRP, and MRP2/4. These specific expression patterns play crucial roles in drug pharmacokinetics, influencing the absorption, distribution, and elimination of drugs.

### Exogenous probes and endogenous biomarkers for drug transporters

Drug transporters play a critical role in the absorption, distribution, and elimination of both substrate drugs and endogenous substances. Their potential impact on pharmacokinetics and drug–drug interactions is an indispensable part of risk assessment in drug application and development. The International Transporter Consortium white papers and regulatory guidelines propose a comprehensive approach (*in vitro, in vivo*, and computational modeling) to evaluate transporter function. These guidelines incorporate the use of exogenous probes and endogenous biomarkers to support rational drug use, predict drug–drug interactions, and assess the inhibitory effects of new molecular entities on transporters [23]. Similar to exogenous probes for drug metabolizing enzymes, low-dose probe drugs can be used to monitor changes of absorption rate, distribution characteristics, and elimination mediated by transporters *in vivo*, thereby providing information about transporter activity. Exogenous probes allow for short-term monitoring of transporter activity following administration. In contrast, endogenous probes offer a broader perspective on transporter function, reflecting their activity across various physiological states over a longer duration.

#### OAT1/3

OAT1 and OAT3 are specifically expressed on the basolateral membrane of renal tubular epithelial cells, exhibiting similar expression levels and a broad overlap in their substrates. They predominantly mediate the excretion of organic anion drugs, such as non-steroidal anti-inflammatory drugs, antiviral drugs, antibiotics, and methotrexate [112, 113]. The FDA-recommended exogenous probes for OAT1 include *p*-aminohippuric acid, adefovir, cidofovir, and tenofovir, and for OAT3 they include estrone-3-sulfate, methotrexate, pravastatin, and benzylpenicillin (Table 2). However, estrone-3-sulfate is also a substrate for OATPs, MATEs, NTCP, and BCRP, and methotrexate is a substrate for OATPs, MRP2, BCRP, OCTs, and MATEs, and pravastatin is a substrate for OATPs, MRP2, NTCP, and BCRP. Furosemide is a common probe substrate for both OAT1 and OAT3, with *K*_m_ values of 38.9 and 21.5 μM, respectively, but it can also be transported by MRP2, MRP4, BCRP, OATP1B1, and OATP1B3. Therefore, it is necessary to assess the effect of nonspecific interactions *in vivo* when using these probe substrates to evaluate OAT1/3 function.

**Table 2.** Exogenous probes and endogenous biomarkers for drug transporters.

Transporters	Exogenous probe (*K*_m_, μM)	Endogenous biomarkers (*K*_m_, μM)
OAT1	*p*-Aminohippuric acid (3.9–28), adefovir (23.8–30), cidofovir (30–58), tenofovir (33.8)	Taurine (379), kynurenic acid (496.7), 4-pyridoxic acid
OAT2	5-Fluorouracil (0.0538)	cGMP (88), prostaglandin E2 (0.713), prostaglandin F2α (0.425)
OAT3	Estrone-3-sulfate (2.2–21.2), methotrexate (10.9–76.6), pravastatin (3.3–27.2), benzylpenicillin (13.9–66)	6β-Hydroxyl cortisol, GCDCA-S (64.3), kynurenic acid (382.2), 4-pyridoxic acid
OATP1B1/OATP1B3	Cholecystokinin octapeptide (OATP1B3:3.82–16.5), estradiol-17β-glucuronide (OATP1B1: 2.5–10; OATP1B3: 18.5–24.6), atorvastatin (OATP1B1: 0.761–18.9; OATP1B3: 0.73), pitavastatin (OATP1B1: 0.429–6.7; OATP1B3: 3.25–3.85), pravastatin (OATP1B1: 27–85.7), rosuvastatin (OATP1B1: 0.8–9.31; OATP1B3: 9.8–14.2)	GCDCA-S (OATP1B1: 9.95; OATP1B3: 5.23), CPI (OATP1B1: 0.13; OATP1B3: 3.95), CPIII (OATP1B1: 0.22; OATP1B3: 1.55)
OCT1/2	Metformin (OCT1: 1060–5450; OCT2: 680–3356), TEA (OCT1: 69–566; OCT2: 48–500), ASP^+^ (OCT1: 9.21; OCT2: 24), MPP^+^ (OCT1: 15–25; OCT2: 8–25)	Creatinine (OCT2: 1860–18 800), thiamine (OCT2: 147.2)
MATE1/2-K	Metformin (MATE1: 202–780; MATE2-K: 1050–1980), TEA (MATE1: 220–380; MATE2-K: 760–830), MPP^+^	/
P-gp	Digoxin (73–181), fexofenadine (150), loperamide (11.4), quinidine (10–18.2), vinblastine (0.8–253)	/
PEPT1/2	Cephalexin (PEPT1: 7.97), cefadroxil	Carnosine (PEPT2: 59.4)

Affinity data for exogenous probes is sourced from https://transportal.compbio.ucsf.edu/

OAT1/3 mediate the renal excretion of numerous endogenous substances, such as hippuric acid, 3-carboxy-4-methyl-5-propyl-2-furanpropanoic acid (CMPF), kynurenic acid, 4-ethylphenyl sulfate, indole-3-acetic acid, *p*-cresol sulfate, *N*-(cinnamoyl)glycine, 4-pyridoxic acid, and homovanillic acid [114–116]. Kynurenic acid is a substrate for both OAT1 and OAT3, with *K*_m_ values of 496.7 and 382.2 μM, respectively. The protein binding rate of kynurenine is nearly 100%, making it barely filtered by the glomerulus, and thus it serves as a specific biomarker for assessing OAT1/3 function *in vivo* [117, 118]. In addition, 4-pyridoxic acid can also serve as an endogenous biomarker for evaluating OAT1/3 (Table 2) [119, 120]. Taurine can serve as an endogenous biomarker for OAT1, and 6β-hydroxycortisol and glycochenodeoxycholate-3-sulfate (GCDCA-S) can be used to assess OAT3 (Table 2). However, taurine and 6β-hydroxycortisol/GCDCA-S are mediated by MATE1/2-K and OATPs/NTCP, respectively [121, 122].

#### OAT2

OAT2 is predominantly expressed on the basolateral membrane of hepatocytes and renal proximal tubule epithelial cells, where it mediates the uptake of substrates from the blood into cells. However, its distribution (basolateral or apical membrane) in human renal tubular epithelial cells remains controversial. Although the drugs mediated by OAT2 have not yet been fully elucidated, 5-fluorouracil can serve as an exogenous probe for evaluating OAT2 function, with a *K*_m_ of 0.0538 μM (Table 2) [123].

OAT2 mediates the uptake of endogenous substances such as hippuric acid, cyclic guanosine 3′,5′-cyclic monophosphate (cGMP), prostaglandin E2, and prostaglandin F2α. The transport of cGMP, prostaglandin E2, and prostaglandin F2α is specifically mediated by OAT2, with *K*_m_ values of 88, 0.713 and 0.425 μM, respectively (Table 2). However, the *in vitro* uptake studies of these substrates were conducted using the OAT2 tv1 transcript (NM_006672.3), and it remains unclear whether they can serve as endogenous biomarkers.

#### OATP1B1/1b3

OATP1B1 and OATP1B3 are primarily expressed on the basolateral membrane of hepatocytes and transport a variety of drug substrates, such as fexofenadine and statin drugs, and there is an extensive overlap in the substrates of both OATP1B1 and OATP1B3. OATP1B1 function is positively correlated with the efficacy of statin drugs and negatively correlated with the risk of myopathy. Genetic polymorphisms can significantly affect the function of OATP1B1, with OATP1B1 (rs4149056, 521 T > C) exhibiting low transport activity [5]. The FDA recommends cholecystokinin octapeptide, estradiol-17β-glucuronide, atorvastatin, pitavastatin, pravastatin, and rosuvastatin as exogenous probes to assess their functions (Table 2).

OATP1B1 and OATP1B3 mediate the uptake of endogenous substances such as bile acids, bilirubin, and hormone metabolites. Glycochenodeoxycholate-3-sulfate (GCDCA-S) and coproporphyrin I/III (CPTI/III) can serve as endogenous biomarkers for evaluating OATP1B1 and OATP1B3 functions (Table 2) [23]. Our study demonstrates that azelaic acid can be used as a specific endogenous biomarker for assessing OATP1B3 [124].

#### OCT1 and OCT2

OCT1 and OCT2 are important organic cation transporters in humans, highly expressed on the basolateral membrane of hepatocytes and renal tubular epithelial cells, respectively. They share overlapping substrates and primarily facilitate the uptake of organic cation drugs, including biguanides and platinum-based drugs. The FDA recommends metformin and tetraethylammonium (TEA) as probes for evaluating OCT1 and OCT2 functions. Additionally, 4-(4-dimethylamino)styryl-*N*-methylpyridinium (ASP^+^) and 1-methyl-4-phenylpyridinium (MPP^+^) can also serve as model substrates for both transporters (Table 2).

OCT2 mediates the transport of numerous endogenous substances, such as creatinine, thiamine, tyramine, histamine, agmatine, and 5-hydroxytryptamine. Among these, creatinine and thiamine can serve as endogenous biomarkers for evaluating OCT2 function (Table 2) [125]. In addition, trimethylamine *N*-oxide and *N*1-methylnicotinamide can be used as an endogenous biomarker for evaluating OCT2 function [126, 127].

#### MATE1 and MATE2-K

MATE1 and MATE2-K are primarily expressed on the apical membrane of renal tubular epithelial cells, mediating the efflux of substrates into the urine. MATE1 is also expressed on the bile canalicular side of the human liver. MATE1 and MATE2-K share overlapping substrates with OCT1/2. The FDA recommends metformin, TEA, and MPP^+^ as exogenous probe substrates for evaluating both MATE1 and MATE2-K functions (Table 2).

Although some studies have shown that MATE1 and MATE2-K mediate the uptake of creatinine, with *K*_m_ values > 2 mM, there is still controversy about whether creatinine can serve as an endogenous biomarker for evaluating the functions of MATE1/2-K [128–130].

#### P-gp

P-gp is one of the most important efflux transporters in humans and is widely distributed across various tissues, including the liver, kidney, intestine, blood–brain barrier, and placenta. It mediates the efflux of various drugs, including immunosuppressants, anticancer agents, antivirals, antiepileptics, and antifungals. The FDA recommends digoxin, fexofenadine, loperamide, quinidine, and vinblastine as exogenous probes to evaluate P-gp function (Table 2).

P-gp also mediates the transport of endogenous substances such as steroid hormones, corticosteroids, and β-amyloid [131, 132]. However, whether these substances can serve as endogenous biomarkers for evaluating P-gp function remains unclear.

#### PEPT1 and PEPT2

PEPT1 and PEPT2 mediate the transport of dipeptides and tripeptides in the intestine and kidney, playing an important role in nutrient absorption and drug transport. In the small intestine, PEPT1 facilitates substrate uptake into intestinal epithelial cells. In the kidney, PEPT1 is expressed at low levels, characterized by low affinity and high transport capacity, and is mainly located in the S1 segment of the renal tubules. PEPT2, which is highly expressed in the proximal renal tubules, has high affinity and low transport capacity, and is primarily located in the S2 and S3 segments of the renal tubules. PEPT1 and PEPT2 synergistically mediate the renal tubule reabsorption of substrate drugs mainly, including antibiotics (such as penicillins and cephalosporins) and antiviral drugs (such as ampicillin) [133]. Cephalexin and cefadroxil can serve as exogenous probes for PEPT1 and PEPT2 (Table 2).

Carnosine and 5-aminolevulinic acid can serve as endogenous biomarkers for PEPT1 and PEPT2, with *K*_m_ values of 59.4 and 24 886 μM, respectively. However, 5-aminolevulinic acid is also a substrate of OAT1 (Table 1) [134, 135].

## Strategies for using markers to evaluate drug metabolizing enzyme and transporter functions

**Figure 3.**
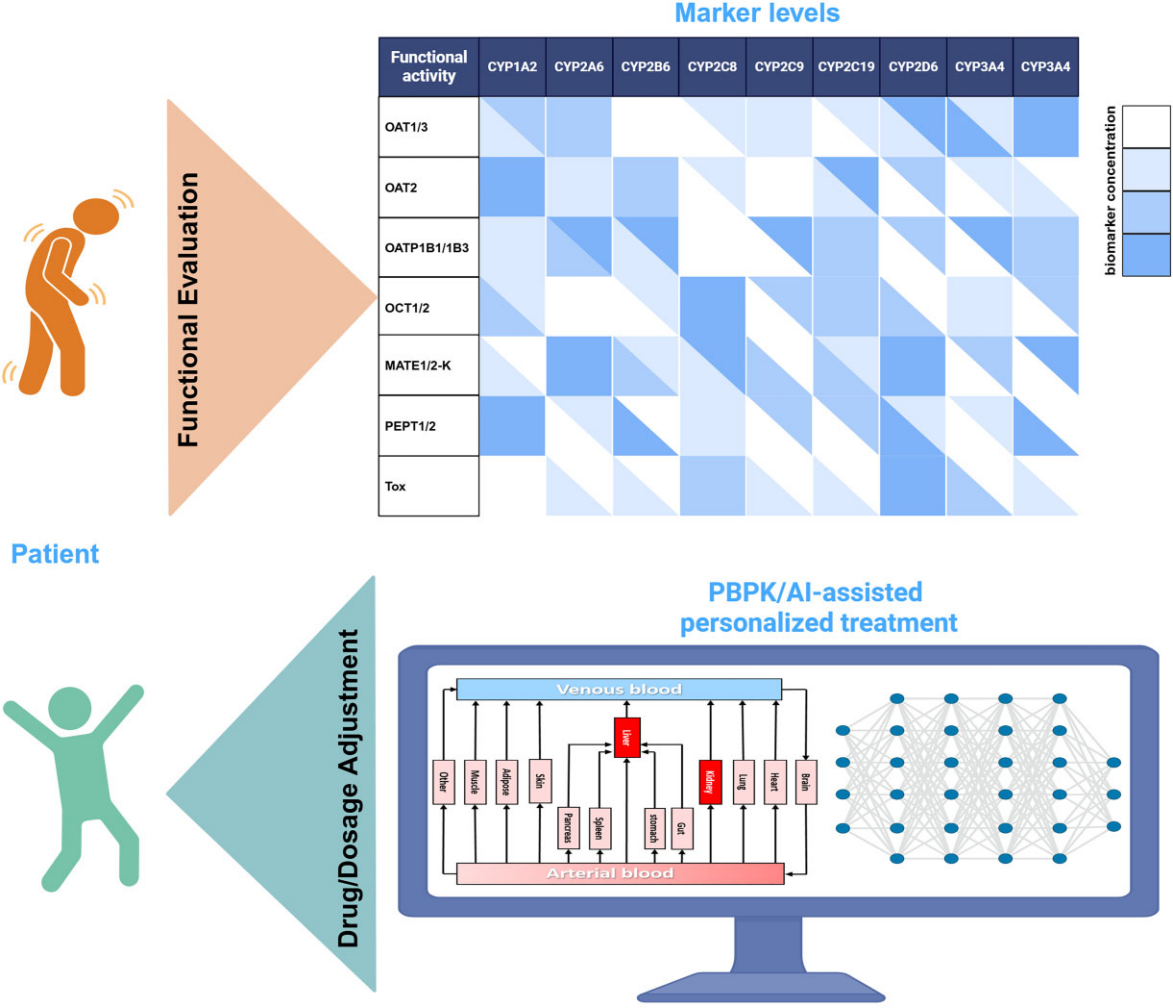
Strategy of drug or dosage adjustment. Drug disposition capacity can be assessed by measuring marker levels of drug metabolizing enzymes and transporters in patient serum and developing PBPK or AI modeling based on marker levels.

Exogenous probes and endogenous biomarkers can accurately reflect the activity of CYPs and transporters under different disease or physiological conditions. Developing multi-probes (cocktail) and multi-biomarker (omics) detection systems can simultaneously reflect the activity of various transporters and metabolic enzymes, uncover compensatory effects and redundancy between pathways, and facilitate the identification of interplay between transporters and metabolic enzymes. However, enormous challenges remain in achieving precise pharmacotherapy, particularly in drug-dose adjustment based on changes in biomarker levels. Currently, these exogenous probes and endogenous biomarkers have not yet been used in clinical practice, possibly because exogenous probes involve ethical concerns and complex procedures that are not widely accepted, and the sensitivity, specificity, and suitability of endogenous biomarkers have not yet been fully elucidated. In the future, physiologically-based pharmacokinetic (PBPK) models and artificial intelligence (AI) based on markers may provide guidance for precision pharmacotherapy in clinical settings (Fig. 3).

### Application of PBPK models

Currently, dose adjustments of drugs based on renal function rely solely on the estimated glomerular filtration rate. However, renal drug excretion involves glomerular filtration, tubular secretion, and reabsorption. In different disease states, changes in glomerular filtration and tubular secretion are not necessarily proportional. Therefore, assessing overall renal clearance using only glomerular filtration markers can be biased [136]. PBPK models simulate anatomical compartments by estimating renal perfusion, filtration, secretion, and reabsorption to predict drug elimination. Granda *et al*. estimated OATs-mediated secretion using kynurenic acid clearance and renal blood flow using isovalerylglycine clearance. They integrated these measurements with glomerular filtration rate into the model to predict renal drug clearance, which improved the accuracy of predicting renal clearance rates for tenofovir and oseltamivir [137]. Therefore, PBPK models based on markers of metabolic enzymes or transporters provide effective strategies for precision pharmacotherapy and drug interaction prediction [138–141].

### Application of AI models

Traditional drug-dose adjustments are typically based on population pharmacokinetic data, which overlooks physiological differences between individuals. AI, through deep learning and large-scale analysis, can identify these differences in drug metabolizing enzymes and transporters from individuals. Markers reflect the dynamic changes in the metabolic and transport functions. By analyzing marker levels in real time, AI can comprehensively predict drug disposition and the risks of drug–drug interactions, providing valuable guidance for precision pharmacotherapy [142–144].

## Conclusion

In recent years, significant progress has been made in precision medicine research based on the functional evaluation of drug metabolizing enzymes and transporters, showing promising potential in personalized therapy and drug development. Although pharmacogenetic testing, therapeutic drug monitoring, and liver and kidney function assessments provide valuable information for precision drug therapy, they also present certain limitations. Existing methods are unable to fully reflect the dynamic changes in the key proteins involved in drug absorption, distribution, metabolism, excretion, and toxicity. In contrast, functional evaluations of metabolic enzymes and transporters allow for a more precise understanding of these core drug-related proteins, enabling the development of optimal therapeutic regimens to improve efficacy and reduce adverse reactions.

At present, research involving exogenous probes for evaluating the functions of metabolic enzymes and transporters is relatively well-established. However, due to ethical concerns, the clinical application of these probes is greatly restricted. In comparison, studies about endogenous biomarkers are, relatively, lagging behind, leading to a lack of effective clinical tools for evaluating the function of these critical proteins. As a result, identifying endogenous biomarkers for the functional evaluation of drug metabolizing enzymes and transporters has become a key task in the field of precision medicine. Furthermore, marker-based PBPK models and AI technologies have already begun to demonstrate potential applications in precision drug therapy. By employing functional marker omics to assess the disposition of drugs *in vivo* and the functional status of relevant drug targets, treatment regimens can be optimized to enhance therapeutic outcomes.

Overall, the study of markers for evaluating the functions of drug metabolizing enzymes and transporters is a crucial direction for advancing precision drug therapy. The integration of PBPK models and AI technologies will further promote the development and application of personalized treatments.
